# Supplementary material for: Novel Driver Strength Index highlights important cancer genes in TCGA PanCanAtlas patients
Source: PeerJ. 2022 Aug 11;10:e13860. doi: 10.7717/peerj.13860 (PMC9375969; doi:10.7717/peerj.13860)
Supplement: Supplemental Information 2 [file peerj-10-13860-s002.zip › COHORTS/genes plots/age/2021_8_16_14_9_distribution_cohorts_45-49.pdf]

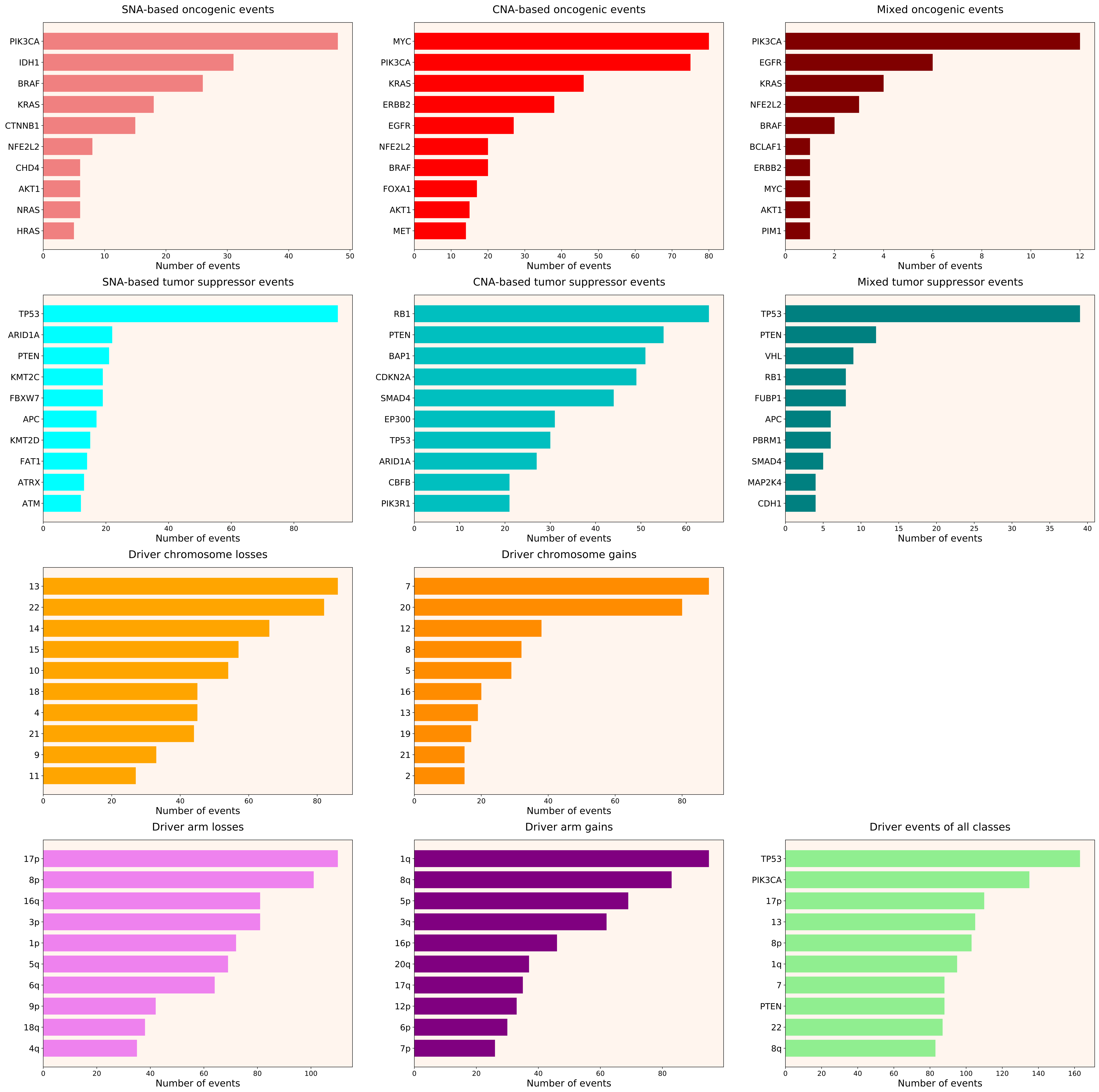

SNA-based oncogenic events
PIK3CA
IDH1
BRAF
KRAS
CTNNB1
NFE2L2
CHD4
AKT1
NRAS
HRAS
0 10 20 30 40 50
Number of events
CNA-based oncogenic events
MYC
PIK3CA
KRAS
ERBB2
EGFR
NFE2L2
BRAF
FOXA1
AKT1
MET
0 10 20 30 40 50 60 70 80
Number of events
Mixed oncogenic events
PIK3CA
EGFR
KRAS
NFE2L2
BRAF
BCLAF1
ERBB2
MYC
AKT1
PIM1
0 2 4 6 8 10 12
Number of events
SNA-based tumor suppressor events
TP53
ARID1A
PTEN
KMT2C
FBXW7
APC
KMT2D
FAT1
ATRX
ATM
0 20 40 60 80
Number of events
CNA-based tumor suppressor events
RB1
PTEN
BAP1
CDKN2A
SMAD4
EP300
TP53
ARID1A
CBFB
PIK3R1
0 10 20 30 40 50 60
Number of events
Mixed tumor suppressor events
TP53
PTEN
VHL
RB1
FUBP1
APC
PBRM1
SMAD4
MAP2K4
CDH1
0 5 10 15 20 25 30 35 40
Number of events
Driver chromosome losses
13
22
14
15
10
18
4
21
9
11
0 20 40 60 80
Number of events
Driver chromosome gains
7
20
12
8
5
16
13
19
21
2
0 20 40 60 80
Number of events
Driver arm losses
17p
8p
16q
3p
1p
5q
6q
9p
18q
4q
0 20 40 60 80 100
Number of events
Driver arm gains
1q
8q
5p
3q
16p
20q
17q
12p
6p
7p
0 20 40 60 80
Number of events
Driver events of all classes
TP53
PIK3CA
17p
13
8p
1q
7
PTEN
22
8q
0 20 40 60 80 100 120 140 160
Number of events
